# Monthly Digest

**Published:** 1889-01

**Authors:** 


### MONTHLY DIGEST.

### The Trend of Dental Thought, as Presented by Our Cotemporaries.

The old saw—"Great minds run in the same channel," suggests itself to the reader of many dental journals. A comparison of the issues of any one month, or consecutive months, will usually show a trend towards one special aspect of dental thought.

For example, run through the journals of the month of December, 1888, and we find the kindred subjects of pulp-devitalization and pulp-capping, the treatment of pulpless teeth, abscesses, etc., and the therapeutic agents employed, with the side-issues. Crowns and bridge work, made the subject of editorials and communications, or discussed in nearly every society meeting reported, to the almost total exclusion of prosthetic dentistry proper, or purely scientific questions.

"In the multitude of counsel there is wisdom," and it is proposed in this, the first number of the present series of papers, to gather together in abbreviated form, with due credit to all parties, both speakers, editors and publishers, the various expressions of thought on this subject,

THE PULP, considered as a unit, as found in several of the dental journals, excluding that which has appeared in the pages of the REGISTER for obvious reasons, these papers being designed for the readers of the REGISTER, who possibly may not have the time to read *all* the other journals.

Taking up the journal which comes first to hand, and which happens to be the *Ohio Journal*, opening almost at random, we find on page 560, a paper which, under a more elaborate title, deals with the subject of

"PULP DEVITALIZATION AND ROOT FILLING."

Dr. Frank Overholser, Logansport, in a paper read before the Indiana State Dental Society, gives his method of treating a tooth which it has been decided to devitalize the pulp. He says the first step is to relieve the pain, for from one to twelve hours; the next, to uncover the pulp well; then he makes an application of arsenious acid, covered by a temporary stopping of gutta purcha, allowed to remain from twelve to twenty-four hours. The thorough removal of the pulp is of the greatest importance. The root is then to be syringed freely with water, wiped out with per-oxide of hydrogen, dried with the hot air syringe, saturated with oil of eucalyptus and powdered idoform, dried again with hot air, and then filled—preferably to the writer with Dawson's cement, which can be mixed thick enough to be handled and well worked into the canal before it sets. If the pulp is found devitalized, with septic conditions present, the canal should be thoroughly disinfected by the use of bi-chloride of mercury, and the napkins or dam adjusted before proceeding as above, with hot air, eucalyptus oil, etc.

Turning to the *Archives*, on page 562, we read that

Dr. J. W. Wick, in a paper read before the St. Louis Dental Society, said that the pulp-capping process is a thing of the past with him ; it is like covering up a volcano when quiet, only to gather force for the coming explosion, which will take place sooner or later. when he anticipates that a tooth will give trouble in the slightest degree after filling, he applies "White's Nerve Paste" at once. The next day he opens into the pulp chamber and removes the pulp from each root, injects with warm water, and applies a dressing of pure carbolic acid on cotton saturated with sandarac. At the end of three days this is removed, the canals again washed with warm water and carbolic acid applied. He then places a small quantity of iodol at the end of each root, and fills with gutta purcha.

PULP-CAPPING.

In the discussion of this paper, Dr. Harper said that he had but little success in pulp-capping. He devitalizes the pulp with *arsenicum album*, mixed with campho-phenique.

Dr. Newby said that he tries to save pulps rather than kill them, and advises more care in the use of arsenic.

Dr. Morrison also takes exception to the want of faith in the vitality of pulps, and advises all to give them a chance.

Dr. Conrad would attempt to save pulps alive in young patients, but anticipates frequent disappointments.

Dr. Prosser also tries to save when there is any prospect of success. When they die it is time to treat and fill.

On page 576 of the same journal we learn that Dr. Shattuck places arsenous acid in an ounce vial, and a little more than covers it with wood creosote. In using this paste, tip the vial so as to take the arsenic from below the creosote, it will stop the most violent toothache in five minutes, where it has been decided to devitalize the pulp.

Dr. Geo. A. Mills, Brooklyn, gives some earnest words of warning as to the USE OF ARSENIC, in pulp-devitalization, and especially of a second application, or its introduction into root-canals. He refers to the many cases cited by Dr. Atkinson, in evidence, and quotes at length from the remarks of Mr. Charles Tomes, in the Odontological Society of London, on the continuity of the human tooth with the rest of the organism. Mr. Tomes spoke of the conclusive researches of Hitzmann and Bodecker showing the vital connection of the cementum with the periosteum, through its protoplasmic net work, which is also continuous at many points with the fibres of the dentine. Speaking of necrosed cement, Mr. Tomes says: "There is much room for surmise, and still more for observation. We do not know in the least what becomes of the protoplasmic contents of the dentinal tubes when a pulp is removed." He suggests its decomposition may poison the cement protoplasm, and that the destructive influence of arsenic may be propagated through the same channels, the alveolo-dental periosteum being in turn diseased, through the decomposing protoplasm of the cement, the dentine of the root being the proximate cause of the mischief.

Taking up the *Independent Practitioner*: page 636, Dr. W. Irving Thayer, Brooklyn, in a paper read before the New Jersey State Dental Society, outlines the system of homeopathic therapeutics in dental pathology, especially in its application to the conservative treatment of exposed pulps. The treatment of freshly exposed pulps, of inflamed pulps, of threatened suppuration, of pulpitis after pulp-capping, etc., and all clearly indicated in the discussion which followed page 647, Dr. Thayer claimed, in reply to questions from Drs. Luckey, Stockton and others, that by the homeopathic system of constitutional treatment, etc., it is possible to save nine out of ten of even highly inflamed pulps, in fact, while admitting that it is a tremendous statement to make, he nevertheless claims *invariable success*. In reply to the suggestion from Dr. Stockton that absolute proof could be had only by opening into the teeth, Dr. Thayer replied that such a demand was unwarrantable when patients are under constant observation. When from frequent examinations and tests, we find that no soreness, aching, or pulpitis supervenes, we may fairly conclude that the pulps are alive, healthy, normal; enjoying perfect physiological action.

Dr. Watkins said that under these very circumstances he had nevertheless, on opening such teeth, found, instead of a beautiful live pulp, with a nice formation of new dentine over it a nasty lot of pus, or a putresent pulp, in almost every case. With him it was almost impossible to save a pulp alive by capping.

The experience of Dr. Ottolengui, and Dr. Stockton in opening teeth with capped pulps, has been similar to that of Dr. Watkins.

Dr. Genese claims great success in pulp-capping, especially in sixth-year molars, using oxide of zinc, carbonate of lime and cocaine. This preparation soothes the pain of an inflamed pulp, excludes moisture, air and pressure, and hardens gradually, taking the form of the pulp. While Dr. Genese would carefully exclude the air, Dr. Luckey agreeing that the microbes in the air are the chief factors in pulp troubles. Dr. Ivory would allow the air in contact with the tissues for a few days as one of the great agents in the healing process.

Dr. Jas. Truman does not think a pulp can be capped and its vitality retained except in the most exceptionally favorable cases.

Dr. W. H. Trueman does not believe that a pulp that has been diseased can be successfully capped by any method whatever; tissue changes have been started that render death inevitable.

Turning back a few pages in the same journal, we find on page 631, that in a paper read by Dr. Wm. H. Potter, Boston, at the October meeting of the Am. Academy of Dental Science, the *surgical principles* underlying the treatment of pulpless teeth are discussed. The teeth being vital organs, and subject to the laws governing the rest of the body, the whole question turns upon the condition of the root canals, and of the peridental tissues. Dr. Potter favors immediate root-filling only in cases of the surgical removal of a live pulp, or of a pulp which has died without exposure to the air or peridental inflammation. Where there is inflammation of the peridental tissues true surgical principles demand the removal of the irritant and the establishment of drainage for the products of inflammation. He considers it bad surgery and bad dentistry to close the outlet afforded by the apical foramen before the disturbance has subsided and the discharge ceased. He does not consider it wise to force the inflammatory products to burrow about in the bony alveolus to effect a passage outwards, thus causing intense pain to the patient. Neither does he deem it safe to use the trephine to effect an artificial outlet, both from the uncertainty of reaching the seat of the abscess and the danger of severing the dental nerve or artery. Where a fistula has already been established the canal may be filled as soon as clean. Root canals he would enlarge, for two reasons: first, the greater facility afforded, and second, the removal of diseased tooth tissue and decomposed substances. Antisepsis he considers essential, relying on peroxide of hydrogen as a disinfectant, alcohol as a detregent and dryer, and carbolic acid and corrosive sublimate as germicides.

On page 524, *The Items of Interest*, we find an article from Dr. Atkinson, on this subject, to which he indicates equal parts of oil of cloves and wood creosote, *kreo-clove* as the most satisfactory disinfectant, with gutta purcha and cholorform for the root-filling. In case of an open fistula the *kreo-clove* is forced through the external orifice, neutralizing any excess with vinegar. Gutta purcha is also forced through sufficiently to form a bulb or button externally, which is allowed to remain until the next day, when it is removed by gentle traction, separating at the weakest point, generally at the apex. If an abscess has proceeded so far as to incite necrosis of the cementum or of the socket, he opens freely into the locality, burs out all dead tissue, and leaves to nature. To abort an abscess, thoroughly incise the gum, periosteum, alveolar plate, peridentium and cementum to the end of the root, opening up the seat of the incipient abscess completely without withdrawing the knife.

In the same journal, page 558, Dr. C. T. Stockwell says that in the treatment of teeth with dead pulps whether with or without fistula, we have to deal with a single agent — sepsis, and nothing short of the solvent and mechanical action of $H^2$ $O^2$ is sufficient. He considers hydrogen peroxide, bi-chloride of mercury, iodoform and eucalyptol as not merely antiseptic, as the word is generally understood, but that they are also *antipyogenic*; that is, they not only destroy the organisms of fermentation and putrification, but they also destroy the pyogenic fungi that are the direct antecedents of pus formation. He considers the non-action of peroxide of hydrogen as sufficient test for the immediate filling of the root-canal with a non-irritant and antiseptic material or combination of materials, one of which shall be iodoform, the ideal antiseptic, the action of which cannot be confined within the pulp canal, but must influence the pyogenic tissues beyond the apical foramen.

If necessary to establish a fistula or gain direct access to the pyogenic tissues, an incision can be made to the bone, without any pain to the patient, by the application of cocaine or pure carbolic acid to the gum.

ALVEOLAR ABSCESS.

Dr. Lankester, London, England, in a paper republished in the same journal (from the *Dental Review*) speaks of the *gum boil* as the external manifestation of an alveolar abscess caused by the decomposition of a dead pulp. The only treatment indicated is the use of iodine before suppuration, and the lancet after, with a warning against external poultices. No treatment of the offending root is referred to other than extraction.

Turning to the *Cosmos*, page 921, we read that:

In the Odontological Society Penn., Dr. Faught gave the history of a case of chronic abscess, in which a number of years previously, the four upper incisors had been accidentally knocked out and immediately washed and reinserted. They began to give trouble after two or three years and three of them were removed one after another. An artificial denture had been worn for sixteen years; abscesses forming and breaking over the remaining incisor every few weeks, when the case came into the hands of Dr. Faught, who removed the last one of the four replanted teeth. The remaining teeth were all firm and sound, and no necrosis to be found after careful examination, yet the fistulae continued to discharge.

Dr. Boice described a similar case in his own practice, and thinks the old abscess sacks had never been broken up.

Dr. Kirk has had similar cases. His treatment is to apply cocaine by hypodermic injection and lay open the parts with a lancet, burring away the affected parts, whether the apex of a root, necrosed bone, or diseased membrane.

Drs. Sudduth and Bennett advise the same mode of treatment.

Dr. Jas. Truman would wait until the sequestrum is thrown off by natural processes.

The questions of the possible formation of a cyst from a pre-existing abscess or pyogenic membrane, and of the formation of pus without the presence of microbes, were discussed by Drs. Kirk, Sudduth, and Tees, the former being considered doubtful, and the latter still an open question.

Dr. Faught related a case in his practice, of an incurable abscess, the tooth having a live pulp, though loose—apparently due to the habit of biting threads.

Affection of the Gums—As we learn from *The Ohio Journal* which republishes the first part of Dr. Lankester's paper on this subject, read before the Students' Society of the National Dental Hospital, London, Eng.

Affections of the gums are broadly classed as local and constitutional, among the former being inflammations from injury, gum-boil or alveolar abscess, simple epulis or fibroma, polypus, or simple hypertrophy, papillary and warty growths, vascular tumors, pyorrhea alveolaris, and primary carcinomata. The constitutional affections, occurring in the course of a general systemic disorder, include syphilis, scrofula, scurvy, stomatitis, aphthae, herpes, and metallic poisons.

Though not closely germane to our main subject we make room for the following, from the same paper.

### EPULIS.

Dr. Lankester describes an epulis as a hard, dense mass of fibrous tissue, starting from the alveolar margin, and spreading under the overlying healthy gum. It usually arises between two teeth, and generally in connection with one, the removal of which together with the excision of the tumor, as a rule, results in immediate cure.

If it returns, it may be necessary to cut away a V-shaped portion of the alveolus together with a very small portion of the subjacent bone. If unrestrained it will continue to enlarge, interfering with mastication and articulation. The tumor is tense and elastic to the touch, of low vascularity, innocent and painless, varying in size from a pea to a walnut. They occur more usually on the labial than on the lingual aspect and twice as frequently in the upper jaw and rarely in an edentulous mouth; if apparently so, a hidden stump will usually be found associated with it.

Closely allied to subjects of Pulpless Teeth and Root Canals, Crown and Bridgework claim our attention.

### THE LOGAN CROWN.

Dr. Ottolengui before the New Jersey State Dental Society as reported in the *Independent Practitioner*, illustrated by drawings and described his methods of setting the Logan Crowns, with amalgam or gutta-percha, and the preparation of the root with his facers and reamers. He also described Dr. Blivan's method of making a Logan Crown from a plate tooth to which he attaches a Logan Crown pin. Dr. Blivan burnishes a piece of platinum over the root, drilling a hole in the centre through which he inserts the pin of the crown; this infringes nobody's patent, and can also be used as a basis for bridgework.

Dr. Ottolengui does not use bands, as a better joint can be made without, and the risk of inflammation of the gums is avoided.

### REMOVABLE PLATE BRIDGES.

The *Cosmos*, in its December issue republishes portions of a chapter from Dr. Evans' recent volume, describing the construction of a removable plate-bridge, in which the root of a superior central incisor is used to support a crown and also the adjoining lateral by the aid of a clasp around the cuspid, the latter being partially squared and the palatal portion notched to form a shoulder on which the clasp rests. In the root of the central a tube is inserted, attached to an iridio-platinum cap which is cemented over the end of the root. The post of the central crown is a split-spring-pivot, which is attached to an outer cap fitting closely over the cap on the root, but which moves easily on and off, the teeth fitting closely against the soft tissues. The method of constructing a similar plate-bridge for the lower teeth is also given.

DIE-PLATE FOR GOLD CROWNS.

Dr. J. W. Whipple, in the *Archives*, gives the following method of making a copper die-plate. A suitable hole is cut in a lead block, which is filled with prepared copper amalgam ; into this, while soft, the crown of a natural tooth is pressed as deep as the cap is to be. When hardened thoroughly, a perfect and sharply-cut impression will be found, sufficiently hard to shape No. 28 pure gold into an absolutely perfect copy of the grinding surface. When no natural crown can be found to suit the articulation of the case in hand, a plaster tooth can be made as follows: a band is adjusted to the root, in which is placed a lump of soft plaster or cement and a bite taken. This is to be carved artistically into a perfect articulating surface and pressed into the copper amalgam in the lead block, as before. The stamping should be done with lead covers, such as are used with the S. S. W. die-plate.

Among the few papers on Operative Dentistry we find the following:

AMALGO-GOLD FILLINGS.

Dr. D. M. Clapp, in a paper read before the N. E. Odontological Society, as reported in the *Cosmos*, describes in detail his method of making large contour fillings of amalgam with a surface finish of gold, in the distal cavities of molars, &c. Defining the good qualities of, and the objections to either gold or amalgam alone, in such cases, Dr. Clapp shows clearly the advantage of combining the two, utilizing the good qualities while getting rid of the imperfections of each.

He emphasizes the importance of thoroughness, neatness, and delicacy in every step of the operation, and also the absolute necessity of a matrix to hold the soft amalgam in place while the gold is inserted. The matrix preferred is of German silver, the thickness of heavy writing paper, long enough to reach one-third round the tooth, the ends being tied together with silk, which is also wound round and round the tooth until the metal is completely covered, giving it strength to resist the pressure of packing, but sufficiently loose to allow an oval burnisher to form the matrix out in the shape required. When the cavity is sufficiently filled with quite dry amalgam, packed solid and dense, Steurer's Plastic Gold is immediately added; the first few pieces will apparently disappear being saturated with mercury, but this will soon be overcome, and the surface can be completed at once with any cohesive gold. No under cuts are required for the retention of the gold, its union with the amalgam being all that is required. The combination gives the color and edge-strength of gold, with the ease of manipulation of amalgam. Thermal and galvanic shocks are less liable to follow its use, and decay less likely to recur than with either material used separately.

In the discussion of Dr. Clapp's paper, Dr. S. E. Davenport described fillings of gold-foil and amalgam, made by anchoring the gold in undercuts on the crown, and built out upon the amalgam and against the matrix, but these fillings had resulted only in failure and dissatisfaction.

Dr. Perry described his method of combination, making the amalgam portion at the first sitting and waiting a day or two before finishing with gold foil.

Dr. Clapp said that he had not been successful in combining the two materials until he had accidentally found the Steurer's Plastic Gold, with which there is never any trouble.

Dr. F. A. Remington said that William's Crystalloid Gold answers well in combination with amalgam.

Dr. Dwinelle gets rid of the excess of mercury in amalgam by heating a small portion over the lamp until the mercury evaporates, and then laying it on the surface of the filling, thus taking up the excess of mercury.

What they think of each other.—"What is the difference between the allopaths and the homœopaths?" asked Mrs. Ceemso of her husband. "Oh," he replied,. "the allopaths think the homœopaths are not ortho-docs."—*Life.*

